# External Stimuli Mediate Collective Rhythms: Artificial Control Strategies

**DOI:** 10.1371/journal.pone.0000231

**Published:** 2007-02-21

**Authors:** Tianshou Zhou, Jiajun Zhang, Zhanjiang Yuan, Anlong Xu

**Affiliations:** 1 School of Mathematics and Computational Sciences, Sun Yat-sen University, Guangzhou, China; 2 State Key Laboratory of Biocontrol and Guangzhou Center for Bioinformatics, School of Life Sciences, Sun Yat-sen University, Guangzhou, China; University of Glasgow, United Kingdom

## Abstract

The artificial intervention of biological rhythms remains an exciting challenge. Here, we proposed artificial control strategies that were developed to mediate the collective rhythms emerging in multicellular structures. Based on noisy repressilators and by injecting a periodic control amount to the extracellular medium, we introduced two typical kinds of control models. In one, there are information exchanges among cells, where signaling molecules receive the injected stimulus that freely diffuses toward/from the intercellular medium. In the other, there is no information exchange among cells, but signaling molecules also receive the stimulus that directionally diffuses into each cell from the common environment. We uncovered physical mechanisms for how the stimulus induces, enhances or ruins collective rhythms. We found that only when the extrinsic period is close to an integer multiplicity of the averaged intrinsic period can the collective behaviors be induced/enhanced; otherwise, the stimulus possibly ruins the achieved collective behaviors. Such entrainment properties of these oscillators to external signals would be exploited by realistic living cells to sense external signals. Our results not only provide a new perspective to the understanding of the interplays between extrinsic stimuli and intrinsic physiological rhythms, but also would lead to the development of medical therapies or devices.

## Introduction

Life is rhythmic. Diverse biological rhythms are generated by thousands of cellular oscillators that are intrinsically diverse but somehow manage to function in a coherent oscillatory state. Physiological functions result from the interactions of cells not only with each other but also with the extracellular medium to generate rhythms essential for life. Experimental works have shown that external stimuli play an important role in the achieving of collective rhythms. Relevant examples include physiological rhythms induced by regular or periodic inputs occurring in the context of medical devices [1], synchronization of electronic genetic networks by an external voltage [2], and diverse regular or irregular rhythms induced by periodic stimuli of a squid giant axon [3]. Another example is that organisms usually display a circadian rhythm in which key processes show a 24-hour periodicity entrained to the light-dark cycle [4], [5]. However, the stimulus-induced essential mechanisms by which the collective rhythm arises remain to be understood.

Although genetic oscillators can be synchronized through appropriate external stimuli, it is important to analyze the effort of the stimuli on intrinsic physiological rhythms since the better understanding of the interactions between the stimuli and physiological rhythms would lead to the development of artificial control strategies and medical devices. However, the wiring of naturally occurring gene regulatory networks would be too complex for qualitative description devoid of mathematics. This complexity has hindered a complete understanding of natural genetic oscillators. Synthetic genetic networks, on the other hand, offer an alternative approach aimed at providing a relatively well controlled test bed in which the functions of natural gene networks can be isolated and characterized in detail [6]. In this direction, the repressilator [7] was recently developed in *Escherichia coli*. Such simple networks represent a first step towards logical cellular control, whereby biological processes can be manipulated or monitored at the DNA level [8]. This control could have a significant impact on post-genomic research [9].

A natural next step in this design effort would be to include the design of artificial control strategies that would be developed to mediate collective rhythms emerging in multicellular structures. In theory, however, even simple control models may show enormous complexity that arises from the interplay between external control amounts and internal dynamics of nonlinear systems [10]–[12]. Therefore, achieving a collective behavior across a population of oscillators by injecting an external substance into the medium must be treated in details and carefully. Here we present theoretical mechanisms of how an external stimulus mediates the collective response by considering two control models: The one is based on the repressilators coupled by quorum sensing where there is an information exchange among cells, and the other on the independent repressilators where there is no information exchange among cells. We show that a signaling molecule that receives a stimulus (or signal) can induce synchronous behaviors across an ensemble of such genetic oscillators, leading to robust collective rhythms in these systems, but also can ruin the achieved collective behaviors. Such a dual function of the signal molecule would be exploited by realistic living cells to sense external signals.

Previous works have indicated that some mechanisms of intercell coupling (e.g., quorum-sensing apparatus [13], [14]) would globally enhance the collective response of a population of genetic oscillators [15]–[17]. However, coupling among oscillators is not, in general, sufficient to achieve synchronization, and many ensembles of coupled oscillators exhibit phase dispersion rather than a synchronized state either because the oscillators actively resist synchronizing [18] or because coupling is too weak or even nonexistent [19]. Using computational modelling, we show that even in the case that the spontaneous synchrony of the individual oscillators cannot be achieved due to the inefficiency of coupling, an appropriate stimulus can compensate such an inefficiency, effectively achieving a collective response.

A recent experimental study [20] has shown that the interplay of gene regulatory networks with population dynamics can lead to the diversity of cell activity that in turn affects (possibly enhances) global behaviors of the entire system due to the effect of noise. Another related study [7] has indicated that extracellular noises arising from changes in cellular environment possibly prevent the observation of macroscopic rhythms in an ensemble of synthetic gene oscillators. Our results indicate that the constrains that local cell oscillators have to face to be noise-resistant could be relaxed in the presence of injecting substances, because a stimulus itself can counteract or suppress noise resistance.

## Analysis

### Case 1: No Information Exchanges Between Cells

#### Model

**Figure 1:**
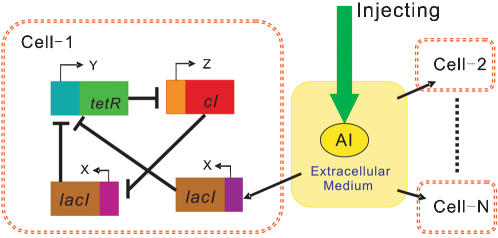
Scheme of a synthetic gene regulatory network in the case of uncoupling.

Accordingly, the “repressilator” network architecture is cyclic [7], in which the protein **LacI** represses the promotor for the *tetR* gene, the **TetR** protein represses the promotor for the *cI* gene, and the **CI** protein represses the promotor for the *lacI* gene. To introduce the external perturbation to each cell, a promoter *P_lac_lux01* that is enhanced by a small molecule **AI**, is also inserted on the repressilator to control another gene *lacI* (Fig. 1).

To model the dynamics of gene expression in the cell population, one must keep track of the temporal evolution of all *mRNA* and protein concentrations from every cell in the network. To describe the behavior of the system, we formulate differential equations in the standard way by ignoring variants in cell density (caused by cell growth and division, for example). The *mRNA* dynamics are governed by
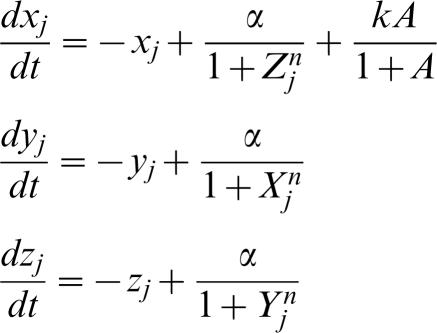
1where *x_j_*, *y_j_* and *z_j_* (here index *j* represents the jth cell. Below is the same) are the concentrations in cell j of *mRNA* transcribed from *lacI*, *tetR* and *cI*, respectively, and the concentrations of the corresponding proteins are represented by X_j_, Y_j_ and Z_j_ (note that the two *lacI* transcripts are assumed to be identical). The concentration of **AI** in the extracellular environment is denoted by A. A certain amount of cooperativity is assumed in the repression mechanism by the Hill coefficient n, where the **AI** activation is chosen to follow a standard Michaelis-Menten kinetics. The model is rendered dimensionless by measuring time in units of the *mRNA* lifetime (assumed equal for all the three genes) and the protein levels in units of their Michaelis constant, i.e., the concentration at which the transcription rate is half its maximal value (also assumed to be equal between all the three genes). The **AI** concentration A is also scaled by its Michaelis constant. α is the dimensionless transcription rate in the absence of repressor, and k is the maximal contribution to *lacI* transcription of saturating amounts of **AI**.

The dynamics of the three proteins are described by the following differential equations:
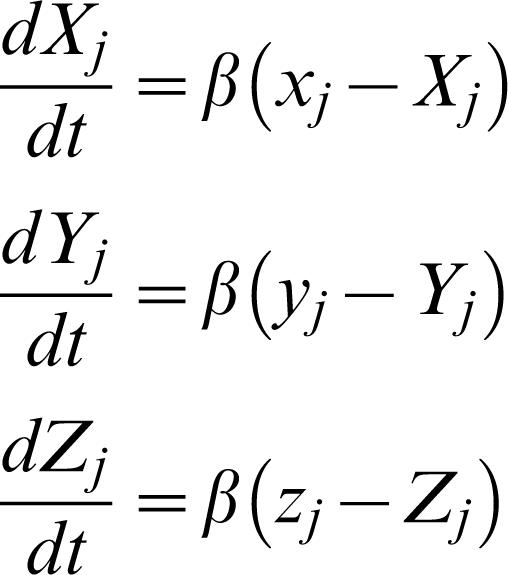
2where the parameter β is the ratio between the *mRNA* and protein lifetimes, and the *mRNA* concentrations have been rescaled by their translation efficiently (proteins produced per *mRNA*, assumed equal for the three genes).

Finally, the dynamical evolution of the extracellular **AI** concentration is governed by
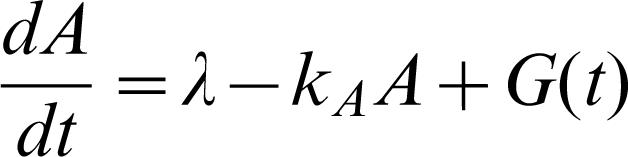
3where λ and k_A_ ares the basal rate of the production and the degradation rate of **AI**, respectively, and *G*(*t*) represents an extracellular stimulus. We will consider the following two types of stimuli:

- Periodic impulsive stimulus: 
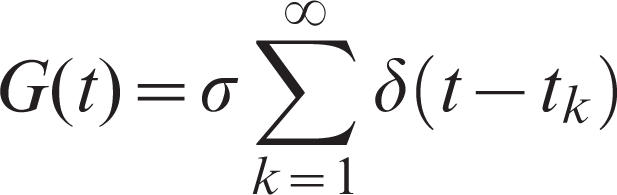
 with *t_k_* = *k*τ, where τ represents the period of impulse. In this case, we assume λ = 0 through this paper;
- Sinusoidal periodic stimulus: *G*(*t*) = σsin(ω*t*).

Here σ represents the strength of stimulus in both cases.

In what follows, we will analyze the effect of the external stimulus *G*(*t*) on the collective behavior in Eqs. **1** **–** **3** by fixing parameters throughout this paper: α = 216, *k_A_* = 1.0 and *n* = 2.

#### Results

**Figure 2:**
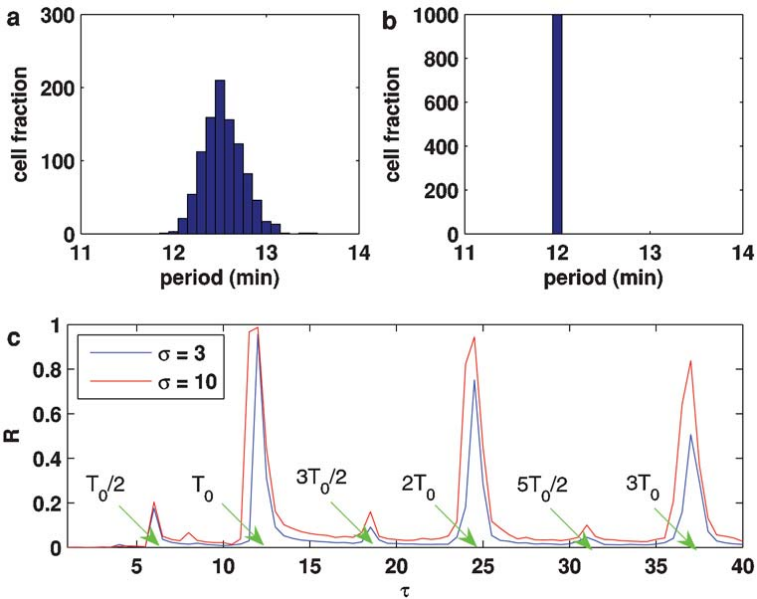
Period histogram (a and b) and dependence of R on τ (c) for 10^3^ cells. (a) k = 0.0, (b and c) k = 2.0. For (b), σ = 10, and for (c), T_0_ = 12.2 (representing the intrinsic period). The lifetime ratio β in the different cells is chosen to obey the Gaussian distribution of mean <β> = 2 and standard deviation Δβ = 0.05.

**Figure 3:**
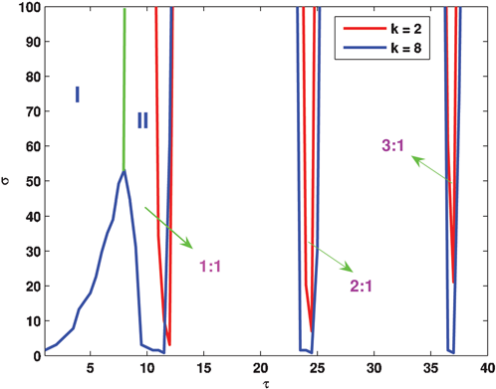
Arnold tongues in the case of periodic impulsive stimulus.

In the hypothetical case of infinite cell dilution (i.e., *k*→0), the system consists of the independent repressilators. Each cell can be approximated as one oscillator with the intrinsic frequency ω_0_≈0.54 for β = 2. On the other hand, in the presence of external stimulus, a new degree of freedom is added to the original six-dimensional phase space to represent dynamics of the signaling molecule governed by Eq. **3**. The resulting system can exhibit synchronized oscillations (Figs. 2 and 3).

The oscillator population will likely contain substantial differences from cell to cell (e.g., extrinsic noise [20]), giving rising to a relatively broad distribution in the periods of the individual oscillators at any given time. In the case of Eqs. **1** **–** **3**, the parameter that affects most markedly the oscillation period is the lifetime ratio β. Accordingly, we model the diversity of a population of cells by considering that β is nonuniformly distributed among the repressilators following a Gaussian law with standard deviation Δβ. The corresponding period distribution of 10^3^ independent cells for Δβ/β = 0.025 is shown in Fig. 2a. With an appropriate periodic impulsive stimulus, perfect locking phase and synchronized oscillation are observed (Fig. 2b). To quantify the degree of synchronization of states of the N oscillators, we introduce an “order parameter” R in the standard way [19]:
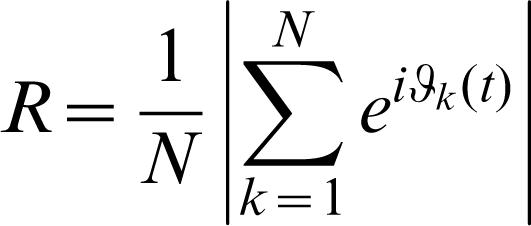
4by using phase ϑ*_k_* of each oscillator, where 
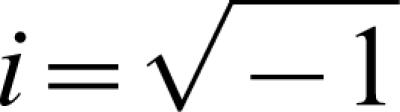
. Then, *R* = 1 corresponds to phase synchronization, whereas *R* = 0 to non-synchronization in the sense of phase. We emphasize that synchronization mentioned in this paper means phase synchronization unless the confusion arises. The dependence relationship between R and τ is shown in Fig. 2c, indicating that within a given time, the synchronization effect is optimal only in the case that the external period is close to the intrinsic period (the average of periods of these individual oscillators is called the intrinsic period of the entire system throughout this paper). In addition, we observe that a population of the oscillators can be entrained to the external periodic driving, forming Arnold tongues similar to those appearing in the case of single oscillator driven by a periodic forcing, as shown in Fig. 3. Refer the detailed explanation and interpretation in the case of coupled noisy oscillators in the next section.

**Figure 4:**
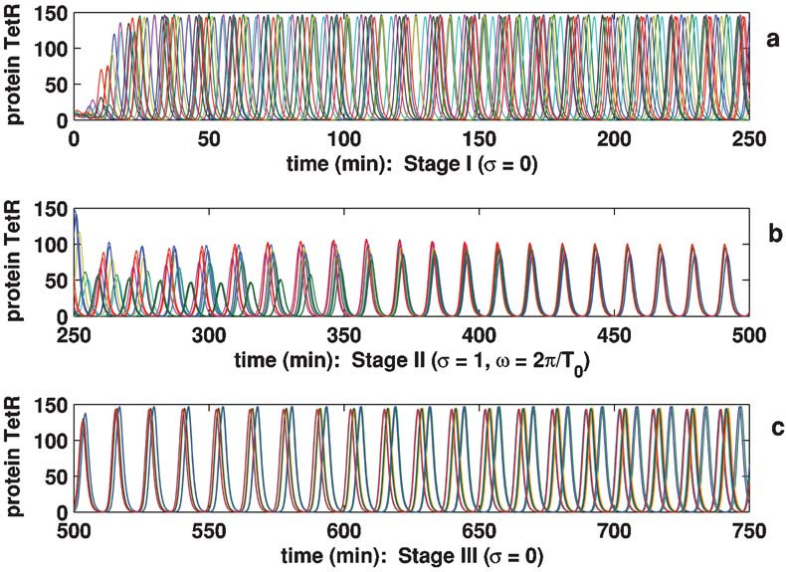
The effect of external sinusoidal stimulus on collective behaviors, where 10^3^ cells are simulated: For k = 0, synchronization cannot be achieved (stages I and III); For k = 2.0, an appropriate external stimulus can induce phase synchronization (stage II). The protein TetR concentrations of 10 cells are plotted. Other parameters are the same as those described in Fig. 2. The initial values of variables in each latter stage take their final values in the right former stage.

On the other hand, in the case of sinusoidal stimulus, the condition under which the external stimulus induces a collective behavior is basically similar to that in the case of periodic impulsive stimulus, that is, only when the extrinsic period is close to an integer multiplicity of the intrinsic period, can the synchronization be achieved. Moreover, the synchronization effect is optimal only in the case that the extrinsic period is equal to the intrinsic period. Fig. 4 shows that an appropriate external stimulus can induce the collective rhythm across an ensemble of cells (see stage II). However, as the external stimulus is removed, the achieved synchronization will be lost (stage III).

### Case 2: Information Exchanges Between Cells

#### Model

**Figure 5:**
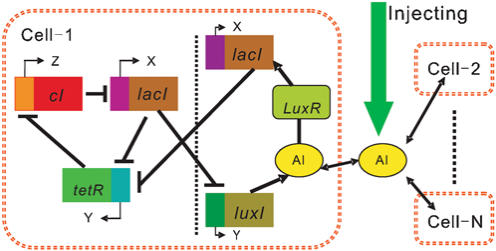
Scheme of the respressilator network coupled to a quorum-sensing mechanism.

Unlike the model considered in **Case 1**, the following model is based on the repressilators coupled to a quorum-sensing apparatus. The scheme for gene regulatory networks is shown in Fig. 5. In this case, except for Eqs. **1** **–** **2**, we also need to give the dynamical equations for **AI** inside the cells and in the extracellular medium.

The dynamical evolution of the intracellular **AI** concentration is affected by degradation, synthesis by **LuxI**, and diffusion through the cell membranes toward/from the intercellular medium. Assume that the **TetR** and **LuxI** have equal lifetimes, implying that their dynamics are identical, and hence we may use the same variable to describe these two protein concentrations. Thus, the **AI** rate equation is governed by
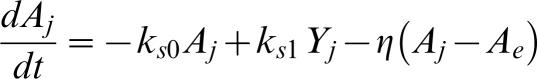
5where η measures the diffusion rate of **AI** across the cell membrane. The parameters *k_s_*_0_, *k_s_*_1_ and η have been made dimensionless by the time rescaling. *A_e_* represents the extracellular concentration of **AI**, the dynamics of which is given by
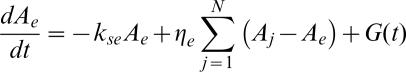
6where η*_e_* stands for the diffusion rate, *k_se_* represents the decay rate of **AI** in the environment, and *G*(*t*) is assumed to be an external stimulus (see **Case 1**). In what follows, we fix parameters: *k_s_*_0_ = 1.0, *k_s_*_1_ = 1.0 and *k_se_* = 1.0, and assume η = η*_e_*. Similar to case 1, we model the diversity of a cell population by considering that β obeys Gaussian distribution with mean <β> = 2 and standard deviation Δβ = 0.05.

#### Results

##### 1. External stimuli can affect the internal oscillations

**Figure 6:**
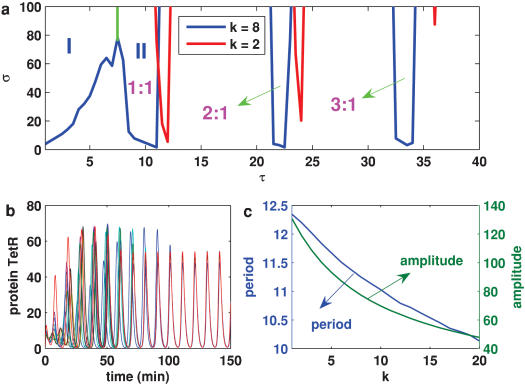
Impulse-induced dynamics in the coupled system governed by Eqs. 1, 2, 4 and 5: 10^3^ cells are simulated. (a) Resonance regions (forming Arnold tongues), where two cases corresponding to k = 2 (labeled by red boundaries) and k = 8 (labeled by blue boundaries), respectively, are displayed; (b) The time evolution of TetRs of 10 cells in resonance region II. η = η*_e_* = 0.1 (a and b), k = 8, σ = 10, and τ = *T*_0_ (b); (c) The effect of parameter k on the mean intrinsic period and amplitude. Other parameters are α = 216 and n = 2, The lifetime ratio β in the different cells is chosen to obey the Gaussian distribution of mean <β> = 2 and standard deviation Δβ = 0.05.

**Figure 7:**
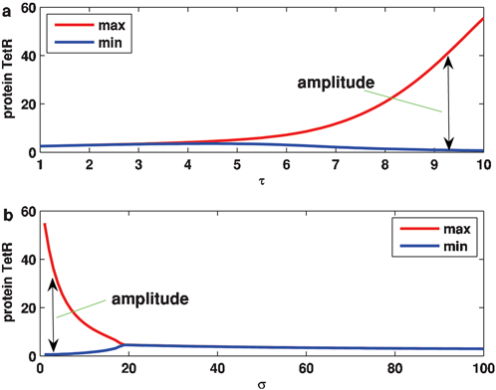
Oscillation death appearing in region I of Fig. 6, where the maximum and minimum of concentration of protein TetR are plotted. (a) σ = 100; (b) τ = 2. Other parameters are the same as those in Fig. 6.

Although there are many interesting properties associated with how an external periodic drive affects a single oscillator (see Ref. [8] and references therein), here we investigate the case of coupled noisy oscillators, focusing on the conditions whereby the periodic impulsive stimulus can cause the dynamics to shift the period and entrain to the external stimulus period (note that the results in the case of sinusoidal stimuli are similar). The boundaries of some major resonance regions that form the so-called Arnold tongues are depicted in the parameter-space plot of Fig. 6a. These regions display a slightly increasing range of the locking period as the strength of stimulus is increased. Without external stimulus (i.e., k = 0), the average period of the autonomous oscillations is T_0_. In the presence of a stimulus, however, the average of intrinsic periods of the individual oscillators depends on parameter k, and moreover the larger the k, the smaller the average period (Fig. 6c). As one might expect, the dominant Arnold tongue (i.e., the first region labeled by II for k = 8 that is near region I) is found around this averaged intrinsic period. Within this resonance region, the period of the oscillations is entrained, and equals the external period. The second largest region of phase locking occurs for periods of stimulus that are an integer multiplicity of the intrinsic period. Inside this Arnold tongue regions (also labeled by region II for k = 8), the synchronization can arise from an arbitrary initial state (Fig. 6b). As a result of the periodic driving, we also observe 2∶1 and 3∶1 lockings, etc, but with the increase of the external driving period, the resonance regions become more and more narrow. Of especial interest is region I labeled in Fig. 6a, where external stimuli can induce rich dynamics, such as oscillation death and synchronization with dampened oscillation [21], [22]. The former is shown in Fig. 7 whereas the latter shown in subsection 3.

##### 2. Compensating the inefficiency of coupling by external stimuli

**Figure 8:**
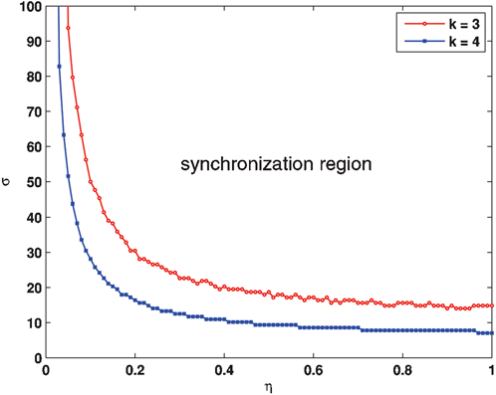
The synchronization region in the σ-η parametric plane, where the impulse period τ is fixed (τ = *T*_0_). Other parameters are the same as those described for Fig. 6.

Fig. 8 shows the dependence relationship between the stimulus strength (σ) and the diffusion rate (η) of **AI** for two different k values, indicating that the synchronization region is enlarged with the increase of k. In particular, within the region in between two curves (the one is labelled by red and the other by blue), the spontaneous synchronization cannot be achieved due to the coupling inefficiency for the fixed k = 3.0 (in this case, even though there are possibly some extra stimuli). On the other hand, for a fixed k, e.g., k = 4.0, the smaller the η, the larger the required σ may be and vice versus, implying that the external stimuli can compensate the inefficiency of such a coupling.

##### 3. Ruining synchronization by external stimuli

**Figure 9:**
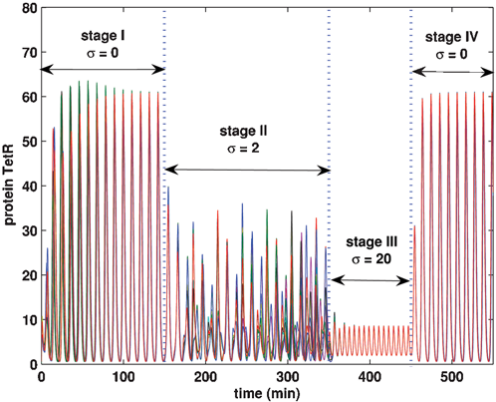
The effect of impulsive stimulus on synchronization of 10^3^ repressilators: Achieving synchronization due to coupling (see stages I and IV); Ruining the achieved synchronization by some external impulses with moderate strength (stage II), and recovering synchronization with the suppressed oscillation amplitude by some strong enough impulses (stage III). The protein TetR concentrations of 10 cells are plotted. k = 20, η = η*_e_* = 3 and τ = 6<*T*_0_. Other parameters are the same as those described for Fig. 6.

Except for inducing synchronization, the external stimuli have also the effect of ruining the achieved synchronization. Fig. 9 shows the process of such a ruin, where four stages are plotted in a way that the initial values of variables in each latter stage take their final values in the right former stage. During stage I (from t = 0 to t = 150 minutes), without the external stimulus, the 10^3^ repressilators achieve synchronization due to coupling; As time t changes from 150 to 350 minutes (stage II), the synchronization is ruined due to the injection of a periodic impulsive stimulus with strength σ = 2; As time is further evolved (see stage III) with the strength of external stimulus σ = 20, the amplitude of oscillations is quickly reduced but synchronization is still observed; Finally, when the external stimulus is removed, the original synchronous behavior is quickly recovered.

##### 4. A new type of synchronization induced by external stimuli

**Figure 10:**
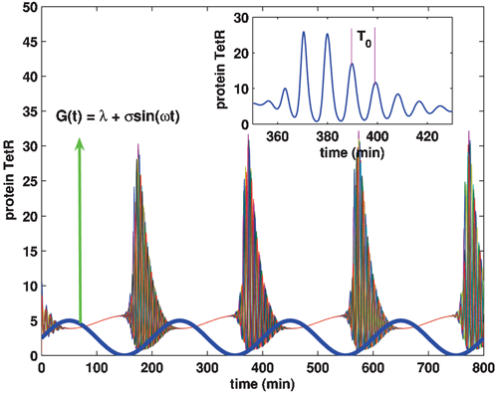
Periodic intermittent synchronization induced by sinusoidal stimulus. The protein TetR concentrations of 10 cells for 10^3^ cells are plotted, where the inset shows a locally enlarged oscillation with period T_0_ and declining amplitudes for one TetR. k = 20, η = η*_e_* = 0.1, λ = 2.5, σ = 2.5 and ω = 2π/200. Other parameters are the same as those described for Fig. 6.

We have previously shown that the external stimuli can enhance/ruin the global behavior. Here we present an interesting synchronization phenomenon induced by the external stimuli, shown in Fig. 10. Different from the usual phase synchrony, it does not appear synchronous within some intervals of time but displays a global synchronous behavior. Such a synchronization looks more a transitional phenomenon appearing in Ref. [23], [24]. Here we call it periodic intermittent synchronization. Furthermore, we present some reasons resulting from the synchronization as follows. When *G*(*t*) = λ+σsinω*t* approaches its maximum, i.e., *G*(*t*) = λ+σ at some ts, the external stimulus induces phase synchronization. It persists until G(t) approaches its minimum. However, there is a period of time from synchronization to unsynchronization or from unsynchronization to synchronization, i.e., synchronization or unsynchronization has “inertia” (see Fig. 2b and d of Ref. [15] and Fig. 5A and B of Ref. [25]), leading that although *G*(*t*)≈0 at some ts, the synchronization is still achieved. With the evolution of time, it will be lost due to the weak external stimulus. The situation is periodically repeated, forming so-called periodic intermittent synchronization.

## Discussion

In this paper, we have proposed two artificial control strategies that are developed to mediate the collective rhythms across an ensemble of genetic oscillators in *Escherichia coli*, and have shown through modelling that can some periodic input substances induce or globally enhance collective rhythms only when the periods of the external stimuli are close to an integer multiplicity of the intrinsic period of the individual oscillators. Such mechanisms of achieving collective rhythms could be exploited by realistic organisms to sense external signals. Our control strategies would also provide guidelines for biological experiments. In particular, the amount and period of a periodic stimulus are independent of state variables of the considered system, so when the stimulus is used to increase the effectiveness of synchronization, we need not measure the state variables at a control instant, thus making the proposed control schemes biologically plausible, and easily being implemented in biological experiments and even by medical uses.

Previous experimental implementations of the repressilator have shown that there is not only substantial variability between cells in the growing population but also a noticeable irregularity in the oscillatory behavior of each individual cell [7]. This irregularity may be caused by noise intrinsic or extrinsic to gene expression [20], [26], [27], plasmid copy-number variability [28], or other unclear external effects. Such a large degree of variability possibly prevents the observation of macroscopic rhythms in a population of synthetic genetic oscillators [7]. Our results have indicated that appropriate external stimuli can counteract the effect of these noises, effectively transforming an ensemble of “sloppy” oscillators into a very reliable collective oscillator.

McMillen *et al* [16] and Garcia-Ojalvo *et al* [15] have shown that intercell signals would globally enhance the collective behaviors across an ensemble of genetic oscillators through a coupling. Our results have demonstrated that appropriate external stimuli can compensate the inefficiency of coupling, effectively achieving a collective response by adjusting the period or amplitude of the periodic stimuli. It would be of interest to investigate the relationship between the strength or period of external stimulus and the strength of coupling, carrying out quantitative curves which predict when a synchronization behavior is achieved.

There are a number of potential applications for the artificial control strategies proposed here. Existing gene therapy approaches typically handle transfected genes that are fixed in either an “on” or “off” state. As our ability to implement cellular control improves, more sophisticated medical interventions may require particular proteins to be expressed on a periodic schedule, and in such ways we would want all cells in a given tissue to operate in a synchronized oscillatory behavior. Furthermore, in probing complex natural networks to deduce their network connectivity, injecting an artificial control amount into the natural system of interest would provide an input whose induced response could provide valuable information about internal cellular processes inside the system, and keeping oscillations synchronous across a population would prevent the introduction of drift in the input signal from cell to cell.
